# Evaluation of hepatic flow changes in early stages after extended hepatectomy by contrast enhanced ultrasound

**DOI:** 10.1186/1470-7330-14-S1-P35

**Published:** 2014-10-09

**Authors:** N Fard, N Rezaei, G Emmami, A Saffari, M Golriz, A Mehrabi, H-P Schlemmer, S Delorme

**Affiliations:** 1 German Cancer Research Center, Department of Surgery, Heidelberg University, Germany

## Background

Extended hepatectomy (EH) is the only curative procedure in patients with large or multi-nodular liver tumours. However, the alterations of the hepatic inflow (HF) after EH and the consequent complications like “small for size syndrome” (SFSS) are still challenging issues. Contrast-enhanced ultrasound (CEUS) is a non-invasive approach to evaluate liver haemodynamics with the advantages of imaging very low blood flow rates at the tissue perfusion level. The aim of this study is to detect the haemodynamic alterations after EH in early stage by CEUS in an experimental setting.

## Method

An in vivo procaine model was studied using a low mechanical index in conjunction with single-level dynamic CEUS. A sulfur hexafluoride contrast agent (SonoVue; Bracco SpA, Milan, Italy) was applied in 5 pigs by intravenous bolus injection. Data were acquired before and after up to 75% sequential liver resections. Corresponding parameters of the time-intensity curve were measured using wash-in/wash-out curve software (Vuebox; Bracco SpA, Milan, Italy).

## Result

Following sequential liver resection, the total HF increased gradually. In detail, the hepatic artery flow decreased 17% and portal vein flow increased around 70% after extended liver resection (75%). Also, with sequential liver resection, the PVP increased gradually up to 33% after extended liver resection (75%).

## Conclusion

Quantitative and qualitative measurement of THF alteration in early stages is feasible by CEUS. CEUS is a suitable modality for follow-up control after EH in order to prevent postoperative complications such as SFSS, which lead to liver failure.

